# Emerging Evidence of the Functional Impact of the miR379/miR656 Cluster (C14MC) in Breast Cancer

**DOI:** 10.3390/biomedicines9070827

**Published:** 2021-07-16

**Authors:** Elan C. McCarthy, Róisín M. Dwyer

**Affiliations:** 1 School of Medicine, Lambe Institute for Translational Research, National University of Ireland Galway, H91 V4AY Galway, Ireland; e.mccarthy14@nuigalway.ie; 2 Science Foundation Ireland (SFI) Research Centre for Medical Devices (CÚRAM), National University of Ireland Galway, H91 W2TY Galway, Ireland

**Keywords:** miR379/miR656 Cluster, C14MC, breast cancer, miR379 family, EMT, TKIs

## Abstract

Many microRNAs exist in clusters that share comparable sequence homology and may target genes in a common pathway. The miR-379/miR-656 (C14MC) cluster is imprinted in the DLK1-Dio3 region of 14q32.3 and contains 42 miRNAs. It plays a functional role in numerous biological pathways including vascular remodeling and early development. With many C14MC miRNAs highlighted as potential tumor suppressors in a variety of cancers, the role of this cluster in breast cancer (BC) has garnered increased attention in recent years. This review focuses on C14MC in BC, providing an overview of the constituent miRNAs and addressing each in terms of functional impact, potential target genes/pathways, and, where relevant, biomarker capacity. Studies have revealed the regulation of key factors in disease progression and metastasis including tyrosine kinase pathways and factors critical to epithelial–mesenchymal transition (EMT). This has potentially important clinical implications, with EMT playing a critical role in BC metastasis and tyrosine kinase inhibitors (TKIs) in widespread use for the treatment of BC. While the majority of studies have reported tumor-suppressing roles for these miRNAs, some have highlighted their potential as oncomiRs. Understanding the collective contribution of miRNAs within C14MC to BC may support improved understanding of disease etiology and present novel approaches to targeted therapy.

## 1. Introduction

First reported by Ambros et al. in 1993 [1], microRNAs (miRNAs) are noncoding RNA sequences of 18–25 nucleotides in length involved in the post-translational regulation of gene expression [1]. miRNAs specifically target the 3′ untranslated region (UTR) of messenger RNAs (mRNAs) via the RNA-induced silencing complex, resulting in post-translational suppression of protein synthesis [2]. These sequences play a critical role in the regulation of numerous physiological and pathophysiological processes [3,4]. In the context of cancer, miRNAs may function as tumor suppressors through binding and repressing oncogenes, or as oncomiRs supporting the progression of cancer through repression of tumor-suppressor genes [4].

It has been suggested that more than half of protein-coding genes contain a binding site for these molecules, with many miRNAs arranged into clusters [2]. miRNA clusters consist of two or more miRNAs which may share comparable sequence homology and may target similar or different genes in a common pathway [5,6,7]. It has been reported that 25% of all miRNAs exist as clusters, with chromosome 19 bearing the largest known human genome cluster [8,9]. In accordance with miRBase, there are a total of 153 clusters containing up to 468 miRNAs in the human genome, which contains almost 2000 precursor miRNAs in its entirety [8]. These clusters have been implicated in many disorders, including cancer, and it has been hypothesized that clustered miRNAs may work synergistically and in a more efficient manner than individual miRNAs [10,11,12].

Following chromosome 19, miR-379/miR-656 (C14MC) is the second largest human miRNA cluster, imprinted in the DLK1-Dio3 region of the 14q32.3 locus. It contains 42 miRNAs and spans approximately 45 kb [2,13]. This cluster plays a functional role in numerous biological pathways and processes including vascular remodeling and early development [14,15]. As the name suggests, miR-379 and miR-656 border each end of C14MC in humans; however, in rodents, this cluster is flanked by miR-379 and miR-410, as it has been hypothesized that murine miR-656 may be nonfunctional [2]. This cluster is regulated by myocyte enhancer factor-2 (MEF2), *JUN* proto-oncogene, and estrogen-related receptor-γ (ESRRG) and has been implicated in the pathogenesis of numerous central nervous system tumors including glioblastoma [11]. The polycistronic nature of C14MC was demonstrated in rat neurons, in which it was found that this cluster was under the positive regulation of MEF2. MEF2 is a transcription factor with a binding site that is highly conserved within mammalian lineages [12]. While transcriptional regulation of this cluster has not been examined in other cancers, MEF2 is highly conserved in humans and so may potentially also play a role in the regulation of C14MC in breast and other cancers.

The C14MC cluster and its associated miRNAs have been extensively studied in a wide variety of cancers, with conflicting reports suggesting that some miRNAs may act as double agents, suppressing cancer in some settings while functioning as oncomiRs in others [16,17]. In particular, the role of this cluster in breast cancer (BC) has garnered increased attention in recent years with a drive towards elucidating the mechanism of action and function of C14MC in this disease setting. This review focuses on C14MC in BC, providing an overview of the constituent miRNAs and addressing each in terms of function (tumor suppressor/oncomiR), potential target genes/pathways, and, where relevant, biomarker capacity.

## 2. MicroRNA-379 Family

Within this cluster, miR-379 belongs to a smaller gene family containing five miRNAs including miR-379, -411, -380, -758, and -1197 [18]. Two members of this subfamily, miR-379 and miR-411, have been investigated in some detail in BC, with all reports suggesting a potentially potent tumor-suppressor function (Table 1) [19,20,21].

With a focus on miRNAs involved in regulation of tumour necrosis factor-beta (TNF-β)-induced interleukin-11 (IL-11), which plays an important role in BC–bone metastases, Pollari et al. [19] identified miR-379 as one of three miRNAs involved in the regulation of TNF-β via targeting of IL-11 [19]. In vitro analysis indicated that miR-379 transfection of cells suppressed the expression of many genes associated with this pathway, e.g., *prostaglandin-endoperoxide synthase 2* (*PTGS2*). Furthermore, these genes were inversely correlated with the expression of genes associated with triple-negative BC (TNBC) [19].

miR-379 has also been a focus of our research group, incorporating in vitro, in vivo, and clinical sample analysis. The data generated supported a potent tumor-suppressor role for this miRNA in BC. In patient samples, levels of miR-379 were significantly reduced in BC tissue compared to tissues from healthy controls, with a further reduction observed in lymph-node metastases of disease [20,21]. Transduction of BC cells with miR-379 resulted in decreased growth and increased necrosis in tumor xenografts. Elevated miR-379 resulted in decreased expression of target proteins cyclin B1 and cyclooxygenase-2 (COX2), which play a critical role in cancer progression [20,21]. Systemic delivery of miR-379 encapsulated in extracellular vesicles (EVs) was also shown to repress tumor growth in vivo [21].

miR-411, also of the miR-379 subfamily of C14MC, has also received attention in the BC setting [22,23]. Employing a panel of BC cell lines of varying subtypes in comparison to nontumorigenic MCF-10A cells, both Guo et al. [22] and Zhang et al. [23] reported lower levels of miR-411 expression in the cancer cells.

Further to this, downregulation of this miRNA was found in patient BC versus matched non-neoplastic tissue, with increased expression associated with grade and evidence of metastasis. In vitro, elevation of miR-411 expression resulted in suppression of BC cell migration, invasion, and proliferation [22,23], potentially mediated through targeting of specific protein 1 (SP1) [22]. In vivo, miR-411 was shown to inhibit progression of TNBC in mice.

Furthermore, it was demonstrated that Ras and growth factor receptor-bound protein 2 (GRB2) activation was involved with and may be a direct target for miR-411 [23].

While it is evident that miR-379 and miR-411 may act as tumor suppressors, other miRNAs in the miRNA subfamily, including miR-380, -758, and -1197, have not been studied in the BC setting. However, their roles in other malignancies have been investigated to some extent. miR-758, for example, has been studied in detail in many cancers, with a potent tumor-suppressor role reported in glioblastoma [44], hepatocellular [45], bladder [46], and papillary thyroid cancers [47].

### Beyond the miR-379 Subfamily: Functional Relevance of C14MC miRNAs in BC

**Figure 1:**
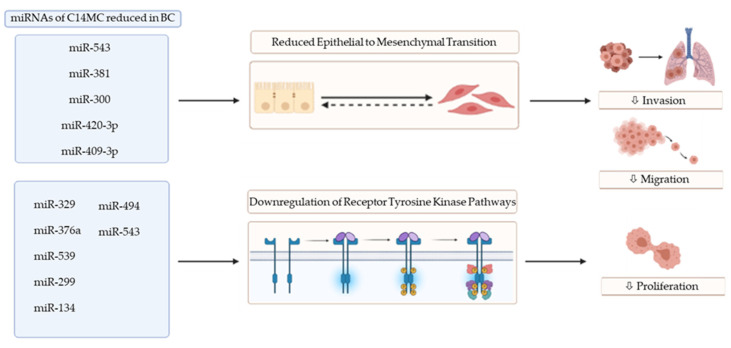
Functional impact of miRNAs of C14MC on epithelial to mesenchymal transition and receptor tyrosine kinase pathways. Abbreviations: BC: breast cancer; ⇩: decreased (image created using Biorender.com—paid subscription. Accessed on 9 July 2021 Biorender 2021).

Outside the miR-379 subfamily, other individual miRNAs of C14MC have been investigated in BC, revealing the regulation of key factors in disease progression and metastasis including tyrosine kinase pathways and factors critical to epithelial–mesenchymal transition (EMT) (Figure 1). This has potentially important implications in the clinical setting, with EMT playing a critical role in BC metastasis and tyrosine kinase inhibitors (TKIs) in widespread use for the treatment of BC (Table 1).

## 3. Regulation of Receptor Tyrosine Kinase (RTK) Pathways

Kang et al. [25] studied the relationship between miR-329 and p130 Crk-associated substrate (p130Cas), an adaptor protein that plays a role in tyrosine-kinase-based signaling in BC. Expression of miR-329 was found to be downregulated in BC patient tissue compared to matched control tissues (*n* = 20), with an inverse correlation with p130Cas expression observed. Analysis of TCGA data of ER-positive BC patients also revealed a significant reduction in miR-329 in BC and improved survival in patients with miR-329-positive tumors. miR-329 was also shown to inhibit classic hallmarks of cancer in vitro and in vivo [25]. In addition, it was determined that the results seen were due to the ability of miR-329 to downregulate p130Cas, with overexpression of this protein resulting in the mitigation of the tumor-suppressor effects seen [25].

miR-539 also appears to play a role in regulation of the tyrosine kinase epidermal growth factor receptor (EGFR) in the BC setting [32]. Levels of miR-539 were found to be markedly reduced in malignant tissue (*n* = 38) versus adjacent nontumorgenic tissue, with levels mirrored in luminal and TNBC cell lines in vitro. Low expression of this miR in clinical samples correlated with metastasis to the lymph nodes [32]. Overexpression of miR-539 abrogated migration and proliferation in vitro, and this was echoed in vivo with attenuation of tumor growth [32]. Further to this, the EGFR was identified as a direct target of this miR, with overexpression of this receptor resulting in the reversal of miR-539 inhibitory effects [32]. Neuropilin-1 (NRP-1) is an isoform-specific receptor for vascular endothelial growth factor (VEGF), and is found in association with diverse RTKs. miR-376a, reported to be significantly downregulated in BC cells, was capable of inhibiting cell proliferation, invasion, and migration, potentially through targeting of NRP-1 [30]. Indeed, overexpression of NRP-1 resulted in abrogation of the inhibitory effects exerted by miR-376a [30]. Expression levels of this miR were significantly downregulated in BC cells, with overexpression resulting in inhibition of cellular proliferation, invasion, and migration. Further to this, Kaplan Meier analyses from two different databases, GSE19783 (*n* = 101) and METABRIC (*n* = 1262) indicated a positive correlation between expression of miR-376a and overall survival of BC patients [30].

miR-299, -134, and -495 have also been implicated in the regulation of serine/threonine kinases and members of the signal transducer and activator of transcription (STAT) family, which are primarily activated by Janus kinases (JAK). miR-299-5p was shown to suppress migration and invasion of BC cell lines in vitro, while overexpression in vivo resulted in inhibition of tumor metastasis in immunocompromised mice [24]. Serine/threonine kinase 39 (STK39) was identified as a potential target of miR-299-5p, with overexpression of STK39 associated with reversal of the inhibitory effects observed [24]. Interestingly, miR-299-5p has also been widely reported as a tumor suppressor in other cancers, including colon [48], hepatocellular [49], lung [50], thyroid [51], and gastric cancers [52].

In two separate studies, miR-134 and miR-495 were shown to play a role in regulation of STATB5 and STAT3, respectively. A comprehensive study by O’Brien et al. [33], which included array analysis of alternations in miRNA expression in triple-negative BC cell lines, found that ten of the miRNAs most substantially downregulated in aggressive cells and their derivative EVs originated from C14MC. Further in vitro analysis revealed an important role for miR-134 in regulation of STATB5, heat shock protein 90 (Hsp90), and B-cell lymphoma 2 (Bcl-2), thus regulating cell proliferation, apoptosis, and response to anticancer therapies. The relevance of miR-134 was further validated using publicly available datasets, where expression of the miRNA was found to be significantly decreased in breast tumors compared to normal breast tissue [33]. Another member of the STAT family with an established role in oncogenesis, STAT-3, was reported to be inversely correlated with miR-495 [34]. Expression levels of miR-495 were also found to be reduced in BC versus adjacent nonmalignant tissue, with levels mirrored in BC cell lines compared to control cells [34]. Treatment with the demethylation agent 5-azacytidine increased expression of miR-495 with a corresponding decrease in STAT-3 and its downstream target vascular endothelial growth factor (VEGF), abrogating cellular function in vitro [34]. Further supporting a tumor-suppressor role for this miR, Guan et al. [35] demonstrated a role for miR-495 in regulation of the RTK human epidermal growth factor receptor 2 (HER2), which plays a key role in BC progression. The team demonstrated that negative regulation of miR-495 by long noncoding RNA lnc SNHG20 supported increased expression of HER2 and promotion of proliferation, invasion, and migration of BC cells in vitro and in vivo [35].

In contrast to the preceding two studies, both Hwang-Verslues et al. [53] and Cao et al. [54] reported a tumor-promoting role for miRNA-495, perhaps due to the specific BC cells/tissues analyzed (Table 2). Upregulation of miR-495 was found in two BC stem cell (BCSC) populations (PROCR^+^/ESA^+^ and CD44^+^/CD24^−low^) and BC cell lines (MDA-MB-468, MDA-MB-361, and SKBR-3), enhancing colony formation in vitro and tumorigenesis in vivo [53]. Furthermore, the miR was reported to directly repress E-cadherin expression, promoting cellular invasion while inhibiting the expression of REDD1 (regulated in development and DNA damage responses 1) and enhancing cell proliferation in hypoxic conditions. Moreover, expression of this miRNA was directly modulated by transcription factor E12/E47, which is highly expressed in BCSCs [53]. Cao et al. [54] found elevated expression of the miRNA in a small number of BC versus nontumor samples (*n* = 7) and identified junctional adhesion molecule A (JAM-A) as a potential target. miR-495 mediated abrogation of JAM-A and resulted in stimulation of BC cell migration in vitro [54].

miR-494 and -543 are other members of the cluster that have been implicated as tumor suppressors in BC by impacting mitogen-activated protein (MAP) kinase signaling [26,27]. In vitro, overexpression of miR-494 in TNBC cells resulted in inhibition of colony formation and metastasis. This effect was mirrored in vivo, with prevention of tumor initiation and migration to the lungs observed with a lower incidence of primary tumor establishment versus the control [26]. In addition, p21-activated kinase 1 (PAK1), an oncogene involved in activation of MAP kinase and MET signaling, was identified as a potential target of miR-494, with an inverse correlation with the miRNA and partial reversal of the tumor-inhibitory effects seen when PAK1 was re-expressed [26].

In relation to miR-543, an initial study by Chen et al. showed that overexpression of the miRNA in BC cells in vitro stimulated cell apoptosis and reduced proliferation. miR-543 was shown to impact the MAP kinase extracellular signal-regulated kinase-2 (ERK-2) pathway, with downstream components of the pathway such as MKS1 and RSK2 reduced when levels of miR-543 were increased [27]. This miRNA is also implicated in the regulation of EMT, as described in the next section.

## 4. Regulation of Epithelial–Mesenchymal Transition (EMT)

miRNAs of C14MC have also been shown to suppress BC progression by targeting factors associated with EMT (Figure 1). Essential during embryonic development, EMT also plays a critical role in breast cancer and other malignancies. This process involves the morphological change of epithelial cells to a more mesenchymal-like composition. This phenotypic change causes a reduction in cell adhesion (e.g., loss of E-cadherin) and increased migration, supporting the metastatic pathway as cells break away from the primary tumor, intravasate, and ultimately invade distant organs [62]. The EMT signature includes increased expression of transcription factors such as TWIST and SNAIL, and a “cadherin switch”, with decreased E-cadherin and increased N-cadherin expression [63,64].

Further investigation of miR-543 revealed a potentially important role in EMT [28]. It was shown that miR-543 could inhibit characteristic traits of EMT and metastasis that had been induced by TGF-β via targeting of zinc finger protein ZNF281 both in vitro and in vivo [28]. In an apparent feedback loop, inc finger protein SNAI1 (SNAIL) and zinc-finger E-box-binding homeobox 1 (ZEB1), EMT-related transcription factors activated by ZNF281, could transcriptionally repress miR-543 [28]. Xue et al. [31] reported downregulated expression of miR-381 in BC tissue (*n* = 27) versus adjacent nontumorgenic tissue, with results also showing reduced expression of this miRNA in BC cell lines [31]. Furthermore, miR-381 expression was shown to have an inverse correlation with cell proliferation, EMT and invasion in vitro. Chemokine (CX-C motif) receptor 4 (CXCR4), which is elevated and associated with EMT and poor survival in many cancers, was identified as a potential target of this miRNA, with a negative correlation observed between the two [31]. miR-300 has also been shown to inhibit TGF-β-induced EMT and to reverse classical characteristics in MDA-MB-231 cells in vitro [65]. Overexpression of this miRNA resulted in suppression of cancer invasion in vitro and metastatic spread in vivo. Furthermore, twist related protein 1 (TWIST) was identified as a potential target of miR-300 [65].

Zhang et al. [39] profiled miR-410-3p expression in a large dataset containing 683 BC and 87 noncancerous breast tissues from the Cancer Genome Atlas (TCGA), and observed a significant downregulation of this miRNA in malignant tissue [39]. In addition, in vitro analysis revealed that overexpression of miR-410-3p in MDA-MB-231 cells attenuated growth, colony formation, and EMT. Furthermore, SNAIL was identified as a direct target, with overexpression of this transcription factor salvaging the effects seen [39]. Endoplasmic reticulum lipid raft-associated 2 (ERLIN2) has also been identified as a target of miR-410 in BC [40]. ERLIN2 is a key driver of cell proliferation, with overexpression of the protein observed in BC cells. Data from this study confirmed ERLIN2 as a direct target, with overexpression of miR-410 shown to downregulate migration, proliferation, and invasion of estrogen receptor (ER)-positive cells in vitro and to attenuate tumor growth in vivo [40]. miR-410 was also shown to be reduced in ER-positive BC versus paired non-neoplastic control tissue (*n* = 15) and displayed an inverse correlation with ERLIN2 [40].

Evidence suggests a divergent function of both mature forms of miR-409 (miR-409-5p and miR-409-3p) in BC, with two studies supporting a tumor-suppressor role for miR-409-3p [41,42] and one demonstrating oncogenic potential of miR-409-5p [61].

Both Ma et al. [41] and Zhang et al. [42] found that expression of miR-409-3p was significantly lower in tumor versus noncarcinogenic breast tissue, with levels mirrored in a panel of BC cell lines compared to nontumorigenic controls. Furthermore, in the larger study of patient samples (*n* = 111) a significant correlation between miR-409-3p levels and poor outcomes amongst BC patients was reported [41]. Increased expression of miR-409-3p was shown to abrogate classic hallmarks of cancer such as migration, invasion, and proliferation in TNBC cells both in vitro and in vivo [41,42]. ZEB1, a transcription factor involved in EMT, and the serine/threonine kinase protein kinase B (AKT1) were identified as targets of miR-409-3p [41,42].

In contrast to the reported role of miR-409-3p, Yu et al. [61] presented evidence of a tumor-promoter role for miR-409-5p, with levels of the miRNA significantly elevated in BC versus non-neoplastic control tissue (*n* = 113). Downregulation of miR-409-5p expression inhibited BC cell migration and proliferation in vitro and attenuated xenograft development in vivo [61]. In addition, Ras suppressor protein 1 (RSU1) was identified as a potential target, with inhibition of miR-409-5p likely to be mediated via inverse upregulation of this protein [61].

While the abovementioned studies highlighted opposing functions of different miRNA isoforms, some miRNAs have been reported to exhibit conflicting functions with papers reporting both tumor-suppressor and oncomiR roles for the same miRNAs (Table 1 and Table 2) [43,56,57]. Lv et al. [43] found that miR-655 was reduced in malignant versus nontumorigenic adjacent matched tissues (*n* = 135), with the level of expression linked to hormone-receptor expression and evidence of metastasis. This was mirrored in cell lines, with basal-like cells expressing much lower levels of miR-655 in comparison to luminal subtypes and a corresponding inhibition of tumor growth and metastatic spread observed in vivo. Furthermore, overexpression of this miRNA abrogated invasion and migration of cancerous cells in parallel with augmentation of cytokeratin and reduced vimentin levels [43]. In addition to this, a negative correlation was observed between miR-655 and paired related omeobox 1 (Prrx1), an EMT inducer with both invasive and migratory characteristics. Overall, it was shown that miR-655 could act as a potential tumor suppressor via interaction with Prrx1 in the BC setting [43]. miR-655 has also been implicated as an oncogenic miRNA. As reported by Majumder et al. [56], overexpression of this miRNA in BC cell lines resulted in upregulation of cell migration, proliferation, invasion, EMT, and ability to form spheroids in vitro. On the other hand, knockdown of miR-655 reversed these effects. Furthermore, it was found that ectopic COX-2 expression induced oncogenic miR-655 via E-type prostanoid receptor 4 (EP4) activation [56]. The relationship with EP4 also linked downstream signaling pathways nuclear factor kappa Beta (NFkβ) and extracellular signal-regulated kinase (ERK) to this miRNA. In addition to this, in vivo experimentation demonstrated increased colony formation in the lungs and metastasis to distant organs, e.g., liver and spleen, upon tail-vein injection of the BC cells overexpressing miR-655 [56]. Following on from this work, the same group further examined the role of miR-655 and its relationship with COX-2 [57] and oxidative stress [58] in BC in vitro. Overexpression of miR-655 in ER-positive BC cells gave rise to an increase in VEGF A, COX-2, and lymphatic vessel endothelial hyaluronan receptor-1 (LYVE1), along with increased migration and tubule formation in vitro [57]. This effect was mitigated with the use of EP4 or ERK inhibitors, suggesting involvement of these pathways. Overexpression of miR-655 also caused an increase in the production of reactive oxygen species (ROS) and superoxide (SO) in ER-positive MCF-7 cells [58]. In addition, Thioredoxin Reductase 1 (TXNRD1), an agent involved in sustaining ROS/SO levels, was upregulated in cells overexpressing miR-655, with PBRM1 and TCF21, negative regulators of TXNRD1, identified as direct targets of the miRNA [58].

## 5. Alternative Mechanisms of Tumor Suppression by miRNAs from C14MC

Tan et al. [29] reported decreased expression of miR-654-5p in BC compared to nontumorigenic cells in vitro, with forced overexpression found to inhibit cell invasive capacity and promote apoptosis. Expression of this miRNA was reduced in human BC versus matched non-neoplastic samples (*n* = 110). In addition, decreased expression of miR-654-5p correlated with advanced stage, evidence of metastasis, and poor survival rates [29]. Epithelial stromal interaction 1 (EPSTI1), which plays a role in M1 macrophage polarisation, was inversely correlated with miR-654-5p, indicating its potential as a target [29].

Peroxisome proliferator-activated receptor-gamma coactivator (PGC-1α) plays a key role in the regulation of mitochondrial function and metastatic spread of BC cells. Lou et al. [36] demonstrated that both miR-485-5p and -3p were reduced in BC versus matched non-neoplastic tissue (*n* = 30), with overexpression of these miRNAs resulting in suppression of mitochondrial respiration and attenuation of cellular invasion and migration in vitro. This effect was seen via the direct targeting of PGC-1α. In vivo, miR-485-3p and miR-485-5p were each found to abrogate metastasis in mice injected with TNBC cells [36].

Two studies reported a tumor-suppressor role for miR-154 [37,38]. Xu et al. [37] reported downregulation of miR-154 in a panel of BC cells (luminal and TNBC subtypes) and 36 cancerous and nontumorgenic tissue pairs. In vitro, the authors focused on the impact of this miRNA on MCF-7 cells, with overexpression resulting in the attenuation of cell migration, invasion, and proliferation. Furthermore, an inverse correlation was observed between miR-154 and E2F transcription factor 5 protein (E2F5) in malignant tissue [37]. E2F5 is involved in many cellular processes including initiation of replication, cell cycle control, and DNA synthesis, and has been reported as an oncogene in many cancers including BC. Overall, this study suggested miR-154 may act as a potent tumor suppressor via direct targeting of E2F5 [37]. A study by Pour et al. [38] also supported a tumor-suppressor role for this miRNA, mediated through regulation of Nicotinamide phosphoribosyl transferase (NAMPT). This is a key enzyme in the nicotinamide adenine dinucleotide (NAD) synthesis pathway, with alteration in the pathway linked with BC progression [38]. Results indicated NAMPT as a direct target of miR-154, with an inverse relationship between this miRNA and levels of the enzyme in malignant cell lines [38]. miR-154 was shown to inhibit NAMPT, with a corresponding reduction in cell viability and escalation of apoptosis observed, along with increased sensitivity to doxorubicin [38].

## 6. Alternative Mechanisms of Tumor Promotion by miRNAs from C14MC

In a study by Ma et al. [59], it was found that expression levels of miR-487a were increased in TNBC cell lines compared to luminal subtypes, with TGF-β found to promote the expression of miR-487a. Furthermore, transfection of these cells with an inhibitor of miR-487a attenuated classic hallmarks of cancer and resulted in a significant reduction in vimentin and a corresponding increase in expression of E-cadherin. MAGI2, a key player in the stability of PTEN, was identified as a potential target of miR-487a [59]. A negative correlation between MAGI2 and expression of miR-487a in BC versus matched normal tissue (*n* = 119) was observed, with expression of this miRNA positively associated with lymph-node metastasis [59]. Further to this, downregulation of the miRNA was associated with increased expression of the tumor suppressor PTEN (Phosphatase and TENsin homolog deleted on chromosome 10) and p-PTEN with abrogation of P-AKT expression in vitro. Moreover, NFKβ was linked to miR-487a, with NFKβ causing an increase in the tumor promoting activity displayed by this miRNA [59].

Further evidence of direct regulation of potent tumor suppressors by this cluster has been presented for miR-300 and miR-382-5p, highlighting these three miRNAs as potential oncomiRs, in contrast to many other miRNAs in the cluster (Table 2). Xu et al. [55] examined the role of miR-300 in the adaptation of cancer hallmarks such as invasion and proliferation of BC cells. It was found that this miRNA was significantly upregulated in BC cell lines and in BC tissues (*n* = 50) versus nonmalignant tissues [55]. Increased expression of miR-300 resulted in stimulation of proliferation and advancement of cell cycle in vitro, and increased tumor growth in vivo. The potent tumor suppressor P53 was identified as a target of this miRNA, with the oncogenic function of miR-300 regulating expression of this protein [55]. miR-382-5p has been shown to target the tumor suppressor Ras-related and estrogen-regulated growth inhibitor (RERG), mitigating the inhibitory role it plays in the Ras/ERK pathway [60]. Furthermore, this miRNA promoted several key characteristics of BC progression such as migration, survival, invasion, and colony formation in vitro and metastatic spread in vivo. Clinically, a negative correlation between miR-382-5p and RERG was identified in BC versus control tissues (*n* = 300) with increased expression of this miRNA in malignant samples and an association with poor patient outcome observed [60].

## 7. MicroRNAs of C14MC as Circulating Biomarkers for BC Diagnostics

As outlined in previous studies, levels of expression of miRNAs of the C14MC cluster have been investigated in patient tissue samples and found to be significantly altered in BC, and have been associated with disease characteristics such as lymph-node status and patient prognosis. Although validation in larger patient cohorts is certainly required, initial studies also support the potential of some of these miRNAs as circulating biomarkers of disease. Circulating biomarkers (liquid biopsy) including circulating tumor cells (CTCs), miRNAs, EVs, mRNAs, and proteins in the blood or other biological fluids of patients have the potential to be used for the detection of tumor characteristics [66]. In comparison to tissue biomarkers, examining the profile of circulating blood provides several advantages. Extraction from patients is quick and easy with minimal pain and risk to the individual due to the relatively noninvasive method of testing [67]. In addition, it allows for monitoring of therapeutic response and/or assessment of disease progression in real time, supporting a more personalized approach to cancer therapy [67]. Despite decades of research in this area, there is no robust and clinically validated biomarker for routine detection and monitoring of breast cancer. Although yet to be realized in the clinical context, miRNAs hold immense potential in this setting.

Van Schooneveld et al. [68] investigated the role of miR-411 and 299-5p as potential biomarkers. In this study, a panel of miRNAs were extracted from BC (*n* = 84) and normal tissues (*n* = 8) and, following analysis, mir-411 and -299-5p of C14MCwere among those selected based on fold change. Expression levels of the miRNAs were analyzed in serum samples from BC patients (*n* = 75, including 16 untreated metastatic BC) and healthy control individuals (*n* = 20). It was found that expression levels of both miR-299-5p and miR-411 were significantly reduced in malignant versus normal blood samples, with the most substantial change seen in sera from patients with the highest disease burden (untreated metastatic disease) versus control samples [68]. The potential role of miR-329 as a tissue and serum biomarker has also been investigated in BC. In a paper published by Li et al. [69], levels of miR-329 were reduced in BC tissue samples (*n* = 134) in comparison to healthy controls (*n* = 70), with downregulated expression of this miRNA correlating with stage and development of metastasis and positively associated with miR-329 expression in the serum of these individuals [69]. Furthermore, it was shown that low levels of miR-329 in BC tissue were linked with poor overall survival rates, raising the potential of this miRNA as a prognostic indicator [69].

In another study, following profiling of a panel of miRNAs from plasma of 127 BC patients and 80 healthy volunteers, specific candidate miRNAs including miR-409-3p and miR-376c of C14MC were selected for further analysis by RT-PCR [70]. The levels of both miRNAs were found to be significantly increased in plasma samples of patients with BC, highlighting their potential as diagnostic biomarkers for early detection of BC [70]. The potential of miR-495 as a circulating biomarker of BC has also been raised [71]. Profiling a panel of miRNAs in peripheral blood mononuclear cells (PBMCs) of 60 BC patients and 60 healthy individuals was performed. miR-495 was significantly reduced in the PBMCs of patients with BC and, within this group, it was significantly lower than those with advanced disease (*n* = 13) compared to early-stage (*n* = 32) disease [71].

## 8. Discussion

Microarray analysis of over 1000 samples including 80 BC samples and 80 matched controls highlighted loss of expression of the C14MC cluster in cancer, signifying that it may function as a tumor suppressor as a whole [12]. This led to a lot of interest in the functional significance of the miRNAs in this cluster. This review supports a potentially important role for C14MC in breast cancer, with functional analysis of many targets validated in preclinical models and patient samples. The studies described provide insight into constituent miRNAs and their interactions with target genes/pathways with well-established roles in the disease. Analysis of 21 miRNAs of the cluster is described herein, with 17 reported as tumor suppressors, 6 as oncomiRs, and, within this, miR495 and miR-655 reported as both. Patterns are emerging that show that many miRNAs from this cluster play a functional role in the downregulation of vital factors associated with disease progression and metastatic spread, including EMT and tyrosine kinase pathways. This may have important clinical implications, as expression levels of these miRNAs may act as surrogate indicators of metastatic potential or response/resistance to TKIs.

While divergent results have been reported by different groups for a subset of the miRNAs, these findings may be due to the cell/sample types included in some cases. Due to the limited number of nontumorigenic breast cell lines available, many studies have compared multiple cancer cell lines to only one (MCF10A) control cell line. Breast cancer is a heterogeneous disease, consisting of different epithelial subtypes classified based on hormone-receptor status. When analyzing patient samples, it is critical to have studies that are sufficiently powered to appropriately represent the heterogeneity of the disease. Indeed, some of the data presented suggest that miRNA function may be dependent on subtype, with different roles reported in ER-positive and ER-negative disease. Furthermore, while reports around the potential of circulating miRNAs of C14MC are interesting, bringing these studies together encounters a challenge associated with use of different starting materials (serum/plasma/PBMCs), which is known to impact the level of circulating miRNAs [72]. In order to reach a consensus and determine the true value of these miRNAs in this context, standardization of methodology and starting material will be critical. While it is unlikely that one specific miRNA will provide the sensitivity and specificity required of a biomarker, based on the initial evidence presented, there may be value in longitudinal investigation of panels of miRNAs from this cluster in the circulation of breast cancer patients.

Taken together, these studies present miRNAs from C14MC as having potentially important roles in breast cancer. Elucidating the target genes and pathways and the ultimate functional relevance of these miRNAs individually and in combination will be critical to understanding their true relevance in this disease. Determination of cell-specific expression using in situ hybridisation and similar techniques will support spatial profiling of their expression levels within the tumor microenvironment, as the expression and function of these miRNAs is unlikely to be limited to epithelial tumor cells. Restoring tumor-suppressing miRNAs or analyzing miRNA expression in patients resistant to TKI may be useful when determining choice of treatment strategy, allowing for a more personalized approach. While the studies describe analysis of individual members of the cluster, understanding how these miRNAs may function synergistically may improve understanding of breast cancer etiology and pave the way for development of targeted therapeutics.

## Figures and Tables

**Table 1:** Tumor-suppressor miRNAs of the C14MC in breast cancer.

miRNA	Target	Validated by	In Vitro	In Vivo	Clinical	Findings	Ref
miR-379	IL-11	Luc RA; RT-PCR	✔			Key player in the metastatic process of BC via TGF-β induction of IL-11 in vitro	[19]
	Cyclin B	WB; RQ-PCR; IHC	✔		✔	Reduced levels in BC samples with decrease in proliferation in vitro	[20]
	COX-2	WB; RQ-PCR; IHC	✔	✔		Potent tumor suppressor, reduction in tumor formation and growth in vivo	[21]
miR-411	SPI	Luc RA; WB	✔		✔	Reduced in BC samples, inhibited proliferation, migration, and invasion in vitro	[22]
	GRB2	WB; RT-PCR; Luc RA; IHC	✔		✔	Decreased levels in samples, inhibited proliferation, migration, and invasion in vitro	[23]
miR-299-5p	STK39	Luc RA; WB	✔	✔		Attenuated migration and invasion in vitro, inhibited metastasis in vivo	[24]
miR-329	P130Cas	WB; IHC	✔		✔	Tumor suppressor in vitro, suppressed proliferation, invasion, and migration	[25]
miR-494	PAK1	WB	✔	✔		Inhibited cancer hallmarks in vitro and tumorigenesis and metastasis in vivo	[26]
miR-543	ERK2	Luc RA; WB; RT-PCR	✔			Inhibited BC progression via attenuation of cell proliferation in vitro	[27]
	ZNF281	IHC; WB; RT-PCR; Luc RA	✔	✔		Abrogated EMT and metastasis in vitro and in vivo	[28]
miR- 654-5p	EPSTI1	Luc RA; WB; RT-PCR	✔		✔	Potent tumor suppressor, reduced proliferation and invasion in vitro	[29]
miR-376a	NRP-1	RIP; Luc RA	✔		✔	Decreased proliferation, migration, and invasion in vitro, reduced in BC sample	[30]
miR-381	CXCR4	Luc RA; RT-PCR; WB	✔		✔	Reduced in BC tissue, inhibiting cell proliferation, EMT, and metastasis in vitro	[31]
miR-539	EGFR	Luc RA; RT-PCR; WB	✔	✔	✔	Inhibited migration and proliferation in vitro, reducing tumor growth in vivo	[32]
miR-134	STAT5B	Immunoblotting	✔		✔	Reduced invasion and migration and increased response to anticancer drugs	[33]
miR-495	STAT3	Luc RA	✔		✔	Inhibited BC cell invasion and proliferation, stimulating apoptosis in vitro	[34]
	HER2	Luc RA	✔	✔		Negatively regulated this miR, promoted cancer hallmarks in vitro and in vivo	[35]
miR-485-3p/5p	PGC-1α	WB; RT-PCR; Luc RA	✔	✔	✔	Reduced in BC samples, abrogated migration and invasion in vitro and in vivo	[36]
miR-154	E2F5	WB; RT-PCR; Luc RA	✔		✔	Decreased in BC samples, reduced invasion, migration, and proliferation in vitro	[37]
	NAMPT	WB; RT-PCR; Luc RA	✔			Reduced cell viability, increased apoptosis and potency of doxorubicin in vitro	[38]
miR-410	SNAIL	Luc RA; WB; RT-PCR	✔		✔	Lower levels in BC samples, attenuated cell growth, colony formation, and EMT	[39]
	ERLIN2	Luc RA; WB; RT-PCR; IHC	✔	✔	✔	Reduced in BC tissue, inhibited invasion and proliferation in vitro and in vivo	[40]
miR-409-3p	ZEB1	Luc RA	✔	✔	✔	Decreased in BC tissue, reduced hallmarks of cancer in vitro and vivo	[41]
	AKT1	Luc Ra; WB; RT-PCR	✔	✔	✔	Potent tumor suppressor, inhibited proliferation and invasion in vitro and in vivo	[42]
miR-655	Prrx1	Luc RA; IHC; WB	✔	✔	✔	Reduced in BC samples, inhibited migration, invasion, and EMT	[43]

Abbreviations: Luc RA: luciferase reporter assay; RT-PCR: reverse-transcription polymerase chain reaction; RQ-PCR: real-time quantitative polymerase chain reaction; WB: Western blot; IHC: immunohistochemistry; RIP: RNA immunoprecipitation; clinical: patient samples; ✔: study performed.

**Table 2:** OncomiRs of the C14MC in breast cancer.

miRNA	Target	Validated by	In Vitro	In Vivo	Clinical	Findings	Ref
miR-495	JAM-A	WB; Luc RA	✔		✔	Increased in BC, promoted migration of BC cells in vitro	[54]
	E-Cadherin	Luc RA; Immunoblotting	✔	✔		Stimulated colony formation in vitro and tumorigenesis in vivo	[53]
miR-300	P53	WB; RT-PCR	✔	✔	✔	Upregulated in BC; increased cancer hallmarks in vitro/in vivo	[55]
miR-655	COX2	WB; RT-PCR	✔	✔		Promoted migration, proliferation, and invasion in vitro and in vivo	[56]
	EP4	WB; RT-PCR	✔			Upregulated migration and tubule formation in vitro	[57]
	PBRM1TCF21	Bioinformatics analysisRT-PCR	✔			Increased production of ROS and SO in luminal BC cells in vitro	[58]
miR-487a	MAGI2	Luc RA; RT-PCR; WB	✔			Stimulated cellular EMT, migration, and invasion in vitro	[59]
miR-382	RERG	Luc RA; RT-PCR; WB IHC	✔	✔	✔	Increased hallmarks of cancer in vitro and in vivo	[60]
miR-409-5p	RSU1	Luc RA; RT-PCR; WB	✔	✔	✔	Increased in BC, stimulated cancer progression in vitro and in vivo	[61]

Abbreviations: Luc RA: luciferase reporter assay; WB: Western blot; RT-PCR: reverse-transcription polymerase chain reaction; IHC: immunohistochemistry; Clinical: patient samples; ✔: study performed.

## Data Availability

Not applicable.
